# Assessment of two missense polymorphisms (rs4762 and rs699) of the angiotensinogen gene and stroke

**DOI:** 10.3892/etm.2012.790

**Published:** 2012-01-11

**Authors:** HYUN-KYUNG PARK, MYUNG-CHUN KIM, SUNG-MIN KIM, DAE JEAN JO

**Affiliations:** 1 Departments of Emergency Medicine and; 2 Neurosurgery, School of Medicine, Kyung Hee University Hospital at Gangdong, Seoul, Republic of Korea

**Keywords:** angiotensinogen, haplotype, intracerebral hemorrhage, ischemic stroke, single nucleotide polymorphism

## Abstract

The renin-angiotensin system has an important role in the pathogenesis of stroke. We investigated whether two missense single nucleotide polymorphisms (SNPs; rs4762, Thr207Met, T207M; and rs699, Met268Thr, M268T) of angiotensinogen (AGT; serpin peptidase inhibitor, clade A, member 8) are associated with the development and clinical phenotypes of ischemic stroke (IS) and intracerebral hemorrhage (ICH). We analyzed 197 stroke patients (120 IS and 77 ICH) and 301 control subjects. The patients were classified into subgroups in accordance to the scores of the National Institutes of Health Stroke Survey (NIHSS, <6 and ≥6) and Modified Barthel Index (MBI, <60 and ≥60). Multiple logistic regression models were used to analyze the genotype and allele distributions of each SNP. One of the missense SNPs, rs4762 (T207M) was associated with the development of ICH (P=0.038 in log-additive model and P=0.021 in allele distributions). The T allele frequency of T207M was higher in the ICH group (16.2%) compared with the control group (9.6%). The TC haplotype frequency differed significantly between the ICH and control groups (P=0.014). With regard to clinical features, T207M correlated with the NIHSS scores of the ICH patients (P=0.039 in codominant1, P=0.015 in dominant, P=0.011 in overdominant and P=0.039 in log-additive models). However, the two missense SNPs, rs4762 and rs699, were not associated with IS and its clinical features, including NIHSS and MBI scores. These data suggest that a missense SNP (rs4762, T207M) of the AGT gene may be associated with the development of ICH and contribute to the neurological functional levels of ICH patients.

## Introduction

Environmental and genetic factors contribute to stroke pathogenesis (1–3). A wide range of genetic studies have reported an association between stroke and single nucleotide polymorphisms (SNPs) of certain candidate genes (4–7). During the years 2001–2010, several researchers published studies on the correlation between stroke risk in Korean patients and the SNPs of candidate genes, including neuropeptide Y (8), methylenetetrahydrofolate reductase (NAD(P)H) (9,10), Fc fragment of IgG, low affinity IIa, receptor (CD32) (11), interleukin 1 receptor antagonist (12,13), thromboxane A2 receptor (14), thromboxane A synthase 1 (platelet) (14), phosphodiesterase 4D cAMP-specific (15), chemokine (C-C motif) ligand 5 (16), interleukin 4 (16), fibrinogen β chain (17), apolipoprotein E (18), paraoxonase 1 (19), paraoxonase 2 (19), 5-methyltetrahydrofolate-homocysteine methyltransferase (10), peroxisome proliferator-activated receptor γ (20), Klotho (21), tumor necrosis factor (12), interleukin 1β (12), and Fas (TNF receptor superfamily, member 6) (22).

The renin-angiotensin system (RAS) plays a key role in the regulation of blood pressure and the homeostasis of fluid and electrolytes (23). In brief, angiotensinogen (AGT; serpin peptidase inhibitor, clade A, member 8) is synthesized in the liver. AGT is a circulating substrate from which renin cleaves angiotensin I. Angiotensin I is converted to angiotensin II by angiotensin I converting enzyme (peptidyl-dipeptidase A) 1 (ACE, also known as kininase II). Angiotensin II, a potent pressor substrate, stimulates aldosterone biosynthesis. In the central nervous system (CNS), angiotensin II also stimulates drinking and increases vasopressin and adrenocorticotropic hormone. The RAS is involved in the pathogenesis of stroke (23,24). Several studies have demonstrated associations between gene polymorphisms concerning the RAS and stroke (25–27). Although the RAS may be implicated in the pathogenesis of stroke, the genetic determinants remain unknown. The aim of this study was to assess whether AGT SNPs were associated with the development and clinical features of stroke, specifically ischemic stroke (IS) and intracerebral hemorrhage (ICH) in a Korean population.

## Subjects and methods

### Study subjects

The subjects were stroke patients who visited the Departments of Neurosurgery and Emergency Medicine in the East-West Neomedical Center and Kyung Hee Medical Center (Seoul, Korea). We recruited 120 IS and 77 ICH patients (Table I) and excluded patients with accidental or iatrogenic strokes, transient ischemic attack, brain tumors and congenital brain disorders. Each stroke patient underwent computed tomography and magnetic resonance imaging. We recruited 301 controls through a general health check-up program. Subjects with neurological diseases, ischemic heart diseases, immunological diseases or any severe diseases were excluded. This study was conducted in accordance with the guidelines of the Declaration of Helsinki and approved by the ethics review committee of the Medical Research Institute, School of Medicine, Kyung Hee University. Informed consent was obtained from all subjects. If a stroke patient was incommunicative, we obtained informed consent from a close relative or guardian.

### Subgroups of stroke patients

All stroke patients were classified into one of two clinical subgroups according to the respective results of the National Institutes of Health Stroke Survey (NIHSS) and Modified Barthel Index (MBI). The severity of 13 neurologic symptoms was measured by the NIHSS to evaluate the neurological functional levels of the patient. The quality of 10 general life activities was also measured by the MBI to evaluate the activity of daily living (ADL).

### SNP genotyping

We selected two well-known missense SNPs (rs4762, Thr207Met, T207M; and rs699, Met268Thr, M268T) of the AGT gene (http://www.ncbi.nlm.nih.gov/ SNP; BUILD 132). The previously reported codon numbers of rs4762 and rs699 are 174 and 235, respectively. T207M and M268T refer to a threonine-to-methionine exchange in codon 207 and a methionine-to-threonine exchange in codon 268, respectively. Peripheral blood samples were collected in EDTA or heparin tubes from all subjects. Genomic DNA was extracted using a QIAamp^®^ DNA mini kit (Qiagen, Valencia, CA, USA) and the genotypes of the two SNPs were determined by direct sequencing (Macrogen Inc., Seoul, Korea). We carried out polymerase chain reactions (PCRs) using the sense and antisense primers of each SNP (Table II) under the following conditions: 40 cycles at 94°C for 30 sec, 58°C for 30 sec and 72°C for 30 sec, and 1 cycle at 72°C for 5 min as a final extension step. The PCR products were sequenced using an ABI PRISM 3730xl analyzer (PE Applied Biosystems, Foster City, CA, USA). SeqMan II software (DNASTAR, Inc., Madison, WI, USA) was used to analyze the sequencing data.

### Statistical analysis

SNPStats (http://bioinfo.iconcologia.net/index.php?module=Snpstats), SNPAnalyzer Pro (Istech Corp., Goyang, Korea) and HelixTree (Golden Helix, Inc., Bozeman, MT, USA) were used to determine odds ratios (ORs), 95% confidence intervals (CIs), and P-values adjusting for age and gender as covariables. Hardy-Weinberg equilibrium (HWE) was calculated by the Chi-square test. Multiple logistic regression models (codominant1, codominant2, dominant, recessive and log-additive) were employed to analyze the genetic data. The linkage disequilibrium (LD) block and haplotypes between two SNPs were estimated using Haploview 4.2 (http://www.broadinstitute.org/scientific-community/science/programs/medical-and-population-genetics/haploview). For numbers <5, the P-value was corrected by Fisher’s exact test. Statistical significance was tested using SPSS 15.0 (SPSS Inc., Chicago, IL, USA) and P<0.05 was considered to indicate a statistically significant result.

## Results

### Clinical features of study subjects

The clinical features of the stroke and control subjects are shown in Table I. The control group (n=301) consisted of 163 males and 138 females. The mean age of the controls was 61.1±10.1 (mean ± SD) years. The IS group (n=120) comprised 68 males and 52 females, and the mean age of the IS patients was 65.7±12.1 years. The ICH group (n=77) had a mean age of 56.2±12.5 years, and consisted of 45 males and 32 females (Table I). The mean ages did not differ significantly between the stroke patients (IS+ICH) and control subjects (P>0.05, data not shown), whereas the mean ages of the IS and ICH patients each differed from that of the controls (P<0.05, data not shown). Therefore, we adjusted for age and gender as covariates when obtaining statistical results from the genetic data analyses. The stroke patients were classified into one of two clinical subgroups in accordance with the NIHSS scores (<6 and ≥6) and MBI scores (<60 and ≥60). The numbers of IS and ICH patients with NIHSS scores <6 were 56 and 24, respectively and with NIHSS scores ≥6 were 56 and 49, respectively. The numbers of IS and ICH patients with MBI scores <60 were 70 and 53, respectively, and with MBI scores ≥60 were 25 and 14, respectively (Table I).

### Genetic analysis

Multiple logistic regression analysis with adjustment for age and gender was carried out using the following models: codominant1 (major allele homozygotes vs. heterozygotes), codominant2 (major allele homozygotes vs. minor allele homozygotes), dominant (major allele homozygotes vs. heterozygotes+minor allele homozygotes), recessive (major allele homozygotes+heterozygotes vs. minor allele homozygotes) and log-additive (major allele homozygotes vs. heterozygotes vs. minor allele homozygotes). The genotype and allele frequencies of the two examined SNPs are shown in Tables III and IV. The two missense SNPs (rs4762, Thr207Met, T207M; and rs699, Met268Thr, M268T) of the AGT gene were in HWE in the IS, ICH and control groups (p>0.05, data not shown). The T207M and M268T SNPs were not associated with the development of IS (Table III). As shown in Table IV, one of the missense SNPs (T207M) was weakly associated with the development of ICH (P=0.038; OR, 1.73; 95% CI, 1.04–2.89 in log-additive model). The T allele frequency of T207M was higher in the ICH group (16.2%) than in the control group (9.6%; P=0.021; OR, 1.82; 95% CI, 1.10–3.02). The genotype and allele frequencies of M268T showed no differences between the ICH and control groups (Table IV). These data suggest that the rs4762 (T207M) SNP of the AGT gene may be associated with ICH and the T allele of rs4762 may be a risk factor for the development of ICH.

### Analysis of clinical phenotypes

All stroke patients were divided into clinical subgroups in accordance to the score of the NIHSS (<6 and ≥6) and MBI (<60 and ≥60). As shown in Table V, one of the missense SNPs (T207M) was associated with the NIHSS scores of the ICH patients (P=0.039; OR, 6.33; 95% CI, 1.25–32.13 in codominant1; P=0.015; OR, 4.85; 95% CI, 1.19–19.65 in dominant; P=0.011; OR, 6.09; 95% CI, 1.21–30.55 in overdominant; and P=0.039; OR, 3.01; 95% CI, 0.94–9.62 in log-additive models). The C/T genotype frequency of rs4762 was higher in the NIHSS ≥6 group (32.6%) than in the NIHSS <6 group (8.3%). The T allele frequency of rs4762 was also higher in the NIHSS ≥6 group (20.4%) than in the NIHSS <6 group (8.3%). However, rs699 did not correlate with the NIHSS scores of the ICH group (Table V). Furthermore, rs4762 and rs699 did not correlate with the MBI scores of the ICH group (data not shown). In the IS group, rs4762 and rs699 were not associated with NIHSS and MBI scores, respectively (data not shown). These results suggest that rs4762 may contribute to the neurological functional levels of the ICH patients.

### Linkage disequilibrium and haplotypes

Haploview 4.2 was used to estimate the LD block and haplotypes between rs4762 and rs699 of the AGT gene. The LD block was moderately made between the two tested SNPs (D’=0.707 and r^2^=0.012 in the control group; D’=0.701 and r^2^=0.013 in the IS group; and D’=0.793 and r^2^=0.017 in the ICH group). We also investigated the association between the haplotypes of these two SNPs and stroke. In Table VI, the frequencies of CC, CT, and TC haplotypes are shown to be 0.714–0.717, 0.176–0.178, and 0.101–0.107, respectively. The TC haplotype differed between the ICH group and the controls (P=0.014), but not between the IS group and the controls. These results suggest that the haplotype of rs4762 and rs699 may be associated with the development of ICH.

## Discussion

In the CNS, AGT is mainly distributed in glial cells in close proximity to renin-containing neurons and is also expressed in astrocytes (28,29). The variant of AGT is considered to be a risk factor in various diseases and to be significant in body fluid homeostasis. In the study by Hajjar *et al*(30), which investigated cerebral blood flow regulation, the rs699 SNP (M268T) was associated with vasoreactivity in Caucasians. Buroker *et al*(31) reported that the AGT 268M allele was associated with mountain sickness. Gomez-Gallego *et al*(32) suggested that the C allele of M268T may favor power sports performance. A study by Yugar-Toledo *et al*(33) showed that carriers of the 268T allele were at increased risk for resistant hypertension, particularly if they were older than 50 years. However, Conen *et al*(34) reported that rs699 was not associated with blood pressure progression and incident hypertension in a large cohort of Caucasian women. Underwood *et al*(35) revealed that minor allele carriers of rs699 were associated with significantly decreased level of insulin resistance when assessed with the homeostasis model assessment of insulin resistance. However, Conen *et al*(36) reported a lack of association between rs699 and incident type 2 diabetes. Ahluwalia *et al*(37) demonstrated that the frequencies of T allele and MT/TT genotype in rs699 were higher and associated with increased risk of diabetic nephropathy in Asian Indians. A study by Wang *et al*(38) indicated that rs699 may confer a predisposition to acquired atrial fibrillation in Han Chinese. Freitas *et al*(39) found that the rs699 TT genotype increased coronary artery disease (CAD) risk in subjects with hypertension and dyslipidemia. In addition, Tsai *et al*(40) reported that the effect of the AGT gene haplotype on CAD disease risk increased significantly in females with hypertension. Zakrzewski-Jakubiak *et al*(41) demonstrated that the haplotype between variant allele of rs4762 and variant allele of rs699 was associated with heart failure. However, Renner *et al*(42) reported that the genotypes and haplotypes of rs4762 and rs699 did not correlate with hypertension, CAD and myocardial infarction.

Focusing on stroke, Saidi *et al*(26) reported that rs699 was associated with IS in Tunisians, but rs4762 was not. Um *et al*(27) revealed that the genotype distribution of rs699 differed between Korean IS patients and controls. Brenner *et al*(24) showed that 207M had a weakly protective effect against brain infarction and 268M was strongly associated with 5-year vascular mortality in Caucasian patients. However, Sethi *et al*(43) reported that rs4762 and rs699 were not associated with increased risk for IS in Europeans. These observed discrepancies may result from differences in ethnicities and/or sample sizes. Our results revealed that the genotypes, alleles and haplotypes of rs4762 and rs699 were not correlated with the development of IS in the Korean population. This agrees with results of the study by Sethi *et al*(43) However, our data revealed that rs4762 was associated with the development of ICH and with the NIHSS scores of the ICH group.

The AGT protein (UniProt ID, P01019) comprises 485 amino acids (AAs) with a signal peptide (from 1 to 33 AAs), AGT chain (from 34 to 485 AAs) and several peptides, including angiotensin-1 (from 34 to 43 AAs), angiotensin-2 (from 34 to 41 AAs) and angiotensin-3 (from 35 to 41 AAs) (http://www.uniprot.org/uniprot). Two missense SNPs (rs4762, ACG207ATG, T207M and rs699, ATG268ACG, M268T) are located in the AGT chain. The underlined characters in ACG207ATG and ATG268ACG indicate nucleotide substitution at each site, resulting in threonine-to-methionine and methionine-to-threonine exchanges in codon 207 and codon 268, respectively. In the HapMap (http://www.hapmap.org/; genome build 36) and the SNP database, the C and T allele frequencies in the rs4762 SNP are reported to be 0.891 and 0.109 in Europeans, 0.969–0.959 and 0.031–0.041 in Sub-Saharan Africans, 0.918 and 0.082 in Japanese, 0.919–0.863 and 0.081–0.138 in Chinese and 0.889 and 0.111 in Koreans, respectively. In our control group, the C and T allele frequencies were 0.904 and 0.096, respectively, which are similar to those observed in Asian populations. The T and C allele frequencies in the rs699 SNP were reported to be 0.588 and 0.412 in Europeans, 0.255–0.080 and 0.920–0.745 in Sub-Saharan Africans, 0.163 and 0.837 in Japanese and 0.134–0.279 and 0.866–0.721 in Chinese, respectively. In our control group, the T and C allele frequencies were 0.181 and 0.819, respectively, which are similar to those observed in Japanese individuals. The C allele frequencies of rs699 in Asians are higher than in Europeans. The rs699 SNP shows ethnic differences in its allele distributions. Our results revealed that the TC haplotypes between rs4762 and rs699 were associated with ICH. Considering the ethnic differences in the C allele frequencies of rs699, our results may, in part, explain a medical phenomenon concerning the high ICH incidence in Asians. To the best of our knowledge, this is the first study of whether two missense SNPs (rs4762 and rs699) of AGT are associated with the development and clinical subgroups of stroke (IS and ICH) in a Korean population. We found that a missense SNP rs4762 (T207M) was associated with the development of ICH (P=0.038 in log-additive model, P=0.021 in allele distributions). The T allele frequency of T207M was higher in the ICH group (16.2%) than in the control group (9.6%). Moreover, T207M was related to the NIHSS scores of ICH (P=0.039 in codominant1, P=0.015 in dominant, P=0.011 in overdominant, and P=0.039 in log-additive models). In addition, the TC haplotype between rs4762 and rs699 differed between the ICH and control groups (P=0.014). This study has certain limitations. First, the number of cases is low. In addition, we lacked age-matched samples among the study groups. Additional studies of a large number of cases and/or age-matched populations are required to confirm our results.

The present study suggests that a missense SNP (rs4762, T207M) of the AGT gene may be associated with the development and neurological functional levels of ICH in a Korean population.

## Figures and Tables

**Table I:** Clinical features of study subjects.

Feature	IS	ICH	Control
Total number (n)	120	77	301
Male/female (n)	68/52	45/32	163/138
Age (mean ± SD, years)	65.7±12.1	56.2±12.5	61.1±10.1
NIHSS (score)			
<6	56	24	
≥6	56	49	
MBI (score)			
<60	70	53	
≥60	25	14	

IS, ischemic stroke; ICH, intracerebral hemorrhage; n, number; SD, standard deviation; NIHSS, National Institutes of Health Stroke Survey; MBI, Modified Barthel Index. Stroke patients with inappropriate clinical data were excluded.

**Table II:** Primer sequences for each SNP.

SNP	S/A	Primer sequence (5′-3′)	Size (bp)
rs4762	S	CTCTCTCTATCTGGGAGCCTTG	323
	A	CAATCTTCTCAGCAGCAACATC	
rs699	S	TGTACAGGGCCTGCTAGTGG	369
	A	ACTGGCTGATCTCAGCTACACA	

SNP, single nucleotide polymorphism; bp, base pair; S, sense; A, antisense.

**Table III:** Genotype and allele frequencies of AGT SNPs in IS and control subjects.

SNP	Genotype/allele	Control n (%)	IS n (%)	Model	OR (95% CI)	P-value	Fisher’s exact P-value
rs4762 (T207M)	Genotype						
	C/C	247 (82.1)	91 (75.8)	Codominant1	1.46 (0.85–2.50)	0.15	
	C/T	50 (16.6)	27 (22.5)	Codominant2	1.78 (0.31–10.15)	0.73	0.66
	T/T	4 (1.3)	2 (1.7)	Dominant	1.48 (0.87–2.50)	0.15	
				Recessive	1.65 (0.29–9.41)	0.58	1.00
				Overdominant	1.44 (0.84–2.47)	0.19	
				Log-additive	1.42 (0.89–2.28)	0.15	
	Allele						
	C	544 (90.4)	209 (87.1)		1		
	T	58 (9.6)	31 (12.9)		1.39 (0.87–2.21)	0.16	
rs699 (M699T)	Genotype						
	C/C	196 (65.5)	78 (65.5)	Codominant1	0.99 (0.62–1.58)	0.91	
	C/T	98 (32.8)	38 (31.9)	Codominant2	1.32 (0.29–5.96)	0.58	0.69
	T/T	5 (1.7)	3 (2.5)	Dominant	1.01 (0.64–1.59)	0.98	
				Recessive	1.32 (0.29–5.94)	0.72	0.69
				Overdominant	0.98 (0.62–1.56)	0.94	
				Log-additive	1.03 (0.68–1.56)	0.9	
	Allele						
	C	490 (81.9)	194 (81.5)		1		
	T	108 (18.1)	44 (18.5)		1.03 (0.70–1.52)	0.89	

AGT, angiotensinogen; SNP, single nucleotide polymorphism; IS, ischemic stroke; n, number; OR, odds ratio; CI, confidence interval. P-values were evaluated via logistic regression analyses adjusting for age and gender. The Fisher’s exact P-value was used if the number was <5. SNPs with inappropriate genotype data were excluded.

**Table IV:** Genotype and allele frequencies of AGT SNPs in ICH and control subjects.

SNP	Genotype/allele	Control n (%)	ICH n (%)	Model	OR (95% CI)	P-value	Fisher’s exact P-value
rs4762 (T207M)	Genotype						
	C/C	247 (82.1)	55 (71.4)	Codominant1	1.72 (0.93–3.19)	0.08	
	C/T	50 (16.6)	19 (24.7)	Codominant2	3.10 (0.63–15.17)	0.12	0.13
	T/T	4 (1.3)	3 (3.9)	Dominant	1.83 (1.01–3.30)	0.05	
				Recessive	2.75 (0.57–13.35)	0.22	0.15
				Overdominant	1.66 (0.90–3.07)	0.11	
				Log-additive	1.73 (1.04–2.89)	**0.038**	
	Allele						
	C	544 (90.4)	129 (83.8)		1		
	T	58 (9.6)	25 (16.2)		1.82 (1.10–3.02)	**0.021**	
rs699 (M699T)	Genotype						
	C/C	196 (65.5)	53 (68.8)	Codominant1	0.84 (0.47–1.48)	0.42	
	C/T	98 (32.8)	21 (27.3)	Codominant2	2.45 (0.55–10.86)	0.29	0.38
	T/T	5 (1.7)	3 (3.9)	Dominant	0.91 (0.53–1.58)	0.75	
				Recessive	2.58 (0.59–11.37)	0.23	0.21
				Overdominant	0.81 (0.46–1.43)	0.46	
				Log-additive	1.02 (0.62–1.66)	0.95	
	Allele						
	C	490 (81.9)	127 (82.5)		1		
	T	108 (18.1)	27 (18.5)		0.97 (0.61–1.54)	0.88	

AGT, angiotensinogen; SNP, single nucleotide polymorphism; ICH, intracerebral hemorrhage; n, number; OR, odds ratio; CI, confidence interval. P-values were evaluated via logistic regression analyses adjusting for age and gender. The Fisher’s exact P-value was used if the number was <5. Bold numbers indicate significant associations.

**Table V:** Genotype and allele frequencies of AGT SNPs in ICH subgroups according to NIHSS scores.

SNP	Genotype/allele	NIHSS <6 n (%)	NIHSS ≥6 n (%)	Model	OR (95% CI)	P-value	Fisher’s exact P-value
rs4762 (T207M)	Genotype						
	C/C	21 (87.5)	31 (63.3)	Codominant1	6.33 (1.25–32.13)	**0.035**	**0.039**
	C/T	2 (8.3)	16 (32.6)	Codominant2	1.81 (0.14–23.25)	0.81	1.00
	T/T	1 (4.2)	2 (4.1)	Dominant	4.85 (1.19–19.65)	**0.015**	
				Recessive	1.21 (0.10–15.09)	0.88	1.00
				Overdominant	6.09 (1.21–30.55)	**0.011**	
				Log-additive	3.01 (0.94–9.62)	**0.039**	
	Allele						
	C	44 (91.7)	78 (79.6)		1		
	T	4 (8.3)	20 (20.4)		2.82 (0.91–8.78)	0.07	
rs699 (M699T)	Genotype						
	C/C	16 (66.7)	35 (71.4)	Codominant1	0.62 (0.19–2.00)	0.67	
	C/T	7 (29.2)	12 (24.5)	Codominant2	0.59 (0.05–7.51)	0.94	1.00
	T/T	1 (4.2)	2 (4.1)	Dominant	0.62 (0.20–1.89)	0.40	
				Recessive	0.69 (0.06–8.45)	0.78	1.00
				Overdominant	0.64 (0.20–2.04)	0.46	
				Log-additive	0.69 (0.27–1.72)	0.42	
	Allele						
	C	39 (81.3)	82 (83.7)		1		
	T	9 (18.8)	16 (16.3)		0.85 (0.34–2.08)	0.72	

AGT, angiotensinogen; SNP, single nucleotide polymorphism; NIHSS, National Institutes of Health Stroke Survey; ICH, intracerebral hemorrhage; n, number; OR, odds ratio; CI, confidence interval. P-values were evaluated via logistic regression analyses adjusting for age and gender. The Fisher’s exact P-value was used if the number was <5. Bold numbers indicate significant associations.

**Table VI:** Haplotype analysis of AGT SNPs in stroke and control subjects.

			Stroke		Control			
Patients	Haplotype	Frequency	+	−	+	−	χ^2^	P-value
Intracebral hemorrhage	CC	0.714	102.2	51.8	437.4	164.6	2.369	0.12
	CT	0.176	26.8	127.2	106.6	495.4	0.008	0.93
	TC	0.107	24.8	129.2	55.8	546.2	5.992	**0.014**
Ischemic stroke	CC	0.717	165.6	74.4	437.8	164.2	1.166	0.28
	CT	0.178	43.4	196.6	106.2	495.8	0.022	0.88
	TC	0.101	30	210	55.4	546.6	2.036	0.15

Haplotype comprises rs4762 and rs699. Haplotype distributions in stroke and control subjects were estimated using Haploview 4.2. The bold number indicates a significant association. SNP, single nucleotide polymorphism; AGT, angiotensinogen.

## References

[1] Grysiewicz RA, Thomas K, Pandey DK (2008). Epidemiology of ischemic and hemorrhagic stroke: incidence, prevalence, mortality, and risk factors. Neurol Clin.

[2] van Asch CJ, Luitse MJ, Rinkel GJ (2010). Incidence, case fatality, and functional outcome of intracerebral haemorrhage over time, according to age, sex, and ethnic origin: a systematic review and meta-analysis. Lancet Neurol.

[3] Wolf PA, Belanger AJ, D’Agostino RB (1992). Management of risk factors. Neurol Clin.

[4] Lanktree MB, Dichgans M, Hegele RA (2010). Advances in genomic analysis of stroke: what have we learned and where are we headed?. Stroke.

[5] Ikram MA, Seshadri S, Bis JC (2009). Genomewide association studies of stroke. N Engl J Med.

[6] Somarajan BI, Kalita J, Mittal B, Misra UK (2011). Evaluation of MTHFR C677T polymorphism in ischemic and hemorrhagic stroke patients. A case-control study in a Northern Indian population. J Neurol Sci.

[7] Tao HM, Chen GZ, Lu XD, Chen GP, Shao B (2010). TGF-β1 869T/C polymorphism and ischemic stroke: sex difference in Chinese. Can J Neurol Sci.

[8] Kim NS, Ko MM, Cha MH, Oh SM, Bang OS (2010). Age and sex dependent genetic effects of neuropeptide Y promoter polymorphism on susceptibility to ischemic stroke in Koreans. Clin Chim Acta.

[9] Han IB, Kim OJ, Ahn JY (2010). Association of methylenetetrahydrofolate reductase (MTHFR 677C>T and 1298A>C) polymorphisms and haplotypes with silent brain infarction and homocysteine levels in a Korean population. Yonsei Med J.

[10] Kim OJ, Hong SP, Ahn JY (2007). Influence of combined methionine synthase (MTR 2756A > G) and methylenetetrahydrofolate reductase (MTHFR 677C > T) polymorphisms to plasma homocysteine levels in Korean patients with ischemic stroke. Yonsei Med J.

[11] Kim YS, Yoo JH, Lee BC (2009). Susceptibility for ischemic stroke in Korean population is associated with polymorphisms of the Fc gamma receptor IIA. Blood Coagul Fibrinolysis.

[12] Lee BC, Ahn SY, Doo HK (2004). Susceptibility for ischemic stroke in Korean population is associated with polymorphisms of the interleukin-1 receptor antagonist and tumor necrosis factor-alpha genes, but not the interleukin-1beta gene. Neurosci Lett.

[13] Lee BC, Lee H, Park HK, Yang JS, Chung JH (2010). Susceptibility for ischemic stroke in four constitution medicine is associated with polymorphisms of FCGR2A and IL1RN genes. Neurol Res.

[14] Park SA, Park BL, Park JH (2009). Association of polymorphisms in thromboxane A2 receptor and thromboxane A synthase 1 with cerebral infarction in a Korean population. BMB Rep.

[15] Kim MK, Kim JT, Choi SM (2009). Phosphodiesterase 4D gene and risk of noncardiogenic ischemic stroke in a Korean population. J Korean Med Sci.

[16] Um JY, Kim HM (2009). Polymorphisms of RANTES and IL-4 genes in cerebral infarction. J Mol Neurosci.

[17] Lee SH, Kim MK, Park MS (2008). beta-Fibrinogen gene-455 G/A polymorphism in Korean ischemic stroke patients. J Clin Neurol.

[18] Kang SY, Lee WI (2006). Apolipoprotein E polymorphism in ischemic stroke patients with different pathogenetic origins. Korean J Lab Med.

[19] Shin BS, Oh SY, Kim YS, Kim KW (2008). The paraoxonase gene polymorphism in stroke patients and lipid profile. Acta Neurol Scand.

[20] Lee BC, Doo HK, Ahn SY (2007). Peroxisome proliferator-activated receptor-gamma Pro12Ala polymorphism is associated with the susceptibility to ischemic stroke in Taeeumin classified by Sasang medicine. Neurol Res.

[21] Kim Y, Kim JH, Nam YJ (2006). Klotho is a genetic risk factor for ischemic stroke caused by cardioembolism in Korean females. Neurosci Lett.

[22] Seo JC, Han SW, Yin CS (2002). Evaluation of a Apo-1/Fas promoter polymorphism in Korean stroke patients. Exp Mol Med.

[23] Marciante KD, Bis JC, Rieder MJ (2007). Renin-angiotensin system haplotypes and the risk of myocardial infarction and stroke in pharmacologically treated hypertensive patients. Am J Epidemiol.

[24] Brenner D, Labreuche J, Poirier O, Cambien F, Amarenco P, GENIC Investigators (2005). Renin-angiotensin-aldosterone system in brain infarction and vascular death. Ann Neurol.

[25] Hong SH, Park HM, Ahn JY (2008). ACE I/D polymorphism in Korean patients with ischemic stroke and silent brain infarction. Acta Neurol Scand.

[26] Saidi S, Mallat SG, Almawi WY, Mahjoub T (2009). Association between renin-angiotensin-aldosterone system genotypes and haplotypes and risk of ischemic stroke of atherosclerotic etiology. Acta Neurol Scand.

[27] Um JY, Moon KS, Lee KM (2003). Polymorphism of angiotensin-converting enzyme, angiotensinogen, and apolipoprotein E genes in Korean patients with cerebral infarction. J Mol Neurosci.

[28] Morimoto S, Cassell MD, Beltz TG (2001). Elevated blood pressure in transgenic mice with brain-specific expression of human angiotensinogen driven by the glial fibrillary acidic protein promoter. Circ Res.

[29] Sherrod M, Davis DR, Zhou X, Cassell MD, Sigmund CD (2005). Glial-specific ablation of angiotensinogen lowers arterial pressure in renin and angiotensinogen transgenic mice. Am J Physiol Regul Integr Comp Physiol.

[30] Hajjar I, Sorond F, Hsu YH (2010). Renin angiotensin system gene polymorphisms and cerebral blood flow regulation: the MOBILIZE Boston study. Stroke.

[31] Buroker NE, Ning XH, Zhou ZN (2010). Genetic associations with mountain sickness in Han and Tibetan residents at the Qinghai-Tibetan Plateau. Clin Chim Acta.

[32] Gomez-Gallego F, Santiago C, Gonzalez-Freire M (2009). The C allele of the AGT Met235Thr polymorphism is associated with power sports performance. Appl Physiol Nutr Metab.

[33] Yugar-Toledo JC, Martin JF, Krieger JE (2011). Gene variation in resistant hypertension: multilocus analysis of the angiotensin 1-converting enzyme, angiotensinogen, and endothelial nitric oxide synthase genes. DNA Cell Biol.

[34] Conen D, Glynn RJ, Buring JE, Ridker PM, Zee RY (2008). Association of renin-angiotensin and endothelial nitric oxide synthase gene polymorphisms with blood pressure progression and incident hypertension: prospective cohort study. J Hypertens.

[35] Underwood PC, Sun B, Williams JS (2011). The association of the angiotensinogen gene with insulin sensitivity in humans: a tagging single nucleotide polymorphism and haplotype approach. Metabolism.

[36] Conen D, Glynn RJ, Buring JE, Ridker PM, Zee RY (2008). Renin-angiotensin and endothelial nitric oxide synthase gene polymorphisms are not associated with the risk of incident type 2 diabetes mellitus: a prospective cohort study. J Intern Med.

[37] Ahluwalia TS, Ahuja M, Rai TS (2009). ACE variants interact with the RAS pathway to confer risk and protection against type 2 diabetic nephropathy. DNA Cell Biol.

[38] Wang QS, Li YG, Chen XD (2010). Angiotensinogen polymorphisms and acquired atrial fibrillation in Chinese. J Electrocardiol.

[39] Freitas AI, Mendonca I, Brion M (2008). RAS gene polymorphisms, classical risk factors and the advent of coronary artery disease in the Portuguese population. BMC Cardiovasc Disord.

[40] Tsai CT, Hwang JJ, Lai LP (2009). Interaction of gender, hypertension, and the angiotensinogen gene haplotypes on the risk of coronary artery disease in a large angiographic cohort. Atherosclerosis.

[41] Zakrzewski-Jakubiak M, de Denus S, Dube MP (2008). Ten reninangiotensin system-related gene polymorphisms in maximally treated Canadian Caucasian patients with heart failure. Br J Clin Pharmacol.

[42] Renner W, Nauck M, Winkelmann BR (2005). Association of angiotensinogen haplotypes with angiotensinogen levels but not with blood pressure or coronary artery disease: the Ludwigshafen Risk and Cardiovascular Health Study. J Mol Med (Berl).

[43] Sethi AA, Tybjaerg-Hansen A, Gronholdt ML (2001). Angiotensinogen mutations and risk for ischemic heart disease, myocardial infarction, and ischemic cerebrovascular disease. Six case-control studies from the Copenhagen City Heart Study. Ann Intern Med.

